# Study and evaluation of neovagina epithelium

**DOI:** 10.5935/1518-0557.20210016

**Published:** 2021

**Authors:** Mauri José Piazza

**Affiliations:** 1 Gynecology- Tocogynechology Department, Universidade Federal do Paraná (UFPR), Curitiba, PR, Brazil

**Keywords:** vaginal agenesis, amnion, neovagina, vaginoplasty

## Abstract

**Objective:**

To evaluate the newly formed epithelium that develops following a neovaginoplasty performed with Amniotic Membrane.

**Methods:**

A retrospective study conducted at the University Hospital of the Federal University, in Curitiba, Paraná, Brazil. A group of 33 patients with Vaginal Agenesis, most of them amenorrhoeic, either incapable of or having difficulty to perform sexual activity, were separated in Subgroup A (27 patients) with Mayer-Rokitansky-Kuster-Hauser Syndrome, and Subgroup B (six patients) with Androgenic Insensitivity Syndrome (Morris Syndrome). Intervention: Banister-McIndoe neovaginoplasty was performed using amniotic membrane graft in 33 patients of Subgroups A and B and evaluated 60-90 days later by vaginal epithelium biopsies. Main Outcome Measure(s): Transmission Electronic Microscopy (TEM) performed Biopsies of neovaginal epithelium in 10 patients. In 20 patients, we analyzed the levels of intensity and presence of Estrogenic Receptors.

**Results:**

Vaginal length was measured (vaginometry) before and after surgery. Before surgery, the vagina was absent in 5 patients (15.15%), vaginal length was 1 cm in 19 patients (57.58%) and in 9 patients (27.27%), it was between 2-3 cm. After surgery, all patients had a vaginal length greater or equal to 5 cm and, in 26 patients (78.8%), vaginal length was 7-8 cm. Seven to eight centimeters average neovagina length allowed patients to have a satisfactory sexual activity after all the surgical procedures to dilate, widen and distend the neomucosa lining. The ERs presented different levels of intensity in the three layers of the neovaginal mucosa. TEM analysis of the vaginal neoepithelium obtained from the amniotic membrane graft revealed all the characteristics of a trophic vaginal epithelium.

**Conclusions:**

In a developing country like Brazil, neovaginoplasty with amniotic membrane graft is considered a great option, being an inexpensive, safe, and easy technique, not requiring any special materials. After a few days (60-90 days), or months, a new epithelium and vagina are obtained allowing patients to have proper sexual activity.

## INTRODUCTION

Anomalies in the genital tract cause discomfort, restlessness and physical and psychological disorders. The agenesis of Mullerian structures and urogenital sinus defects may impair or negatively affect the sexual and reproductive life of these patients. Surgical correction combined with appropriate clinical procedures and psychotherapy support is highly recommended. By refining a surgical corrective technique, this study aims to provide and ensure a convenient sex life to the patients with vaginal agenesis. As of many years, we sought to refine our surgical technique using amniotic membrane grafts and dilating methods with rigid molds, to achieve better outcomes. Our goal is to evaluate the complete take of those grafts in the dissected area of the vesicorectal space and its premature transformation into a vaginal mucosa lining. Outcome analyses revealed vaginal neoepithelium, identified by transmission electron microscopy imaging and estrogen cytosolic receptors determination.

## MATERIAL AND METHODS

A group of 33 young patients, 27 with vaginal agenesis (Mayer-Rokitansky-Kuster-Hauser Syndrome), and 6 cases of male pseudo hermaphroditism (Morris Syndrome), that received outpatient care at the Gynecology-Endocrinology Department were selected to take part of this study. Sixty to ninety days before beginning sexual activity, those patients underwent neovaginoplasty by the McIndoe technique ^(McIndoe & Banister, 1938)^, using a graft surgically manufactured of previously collected amniotic membrane. Regarding the age of the patients, 17 (81.9%) were between 16-25 years old, with average age of 21.79 years, a minimum age of 16 years and maximum of 36 years. Complaints recorded on the first patient visit were amenorrhea in 25 patients (75.75%), dyspareunia in 5 patients (15.15%) and absence of vagina in 3 patients (9.10%). Surgical intervention was conducted in 33 patients, including 6 cases of male pseudo hermaphroditism. The level of estrogen receptors (ERs) was not determined for these patients, and they ended up being excluded from the study due to their irregular response to androgen and estrogen. However, the level of estrogen receptors was determined for 20 patients with vaginal agenesis. Vaginal neoepithelium Fragments from 10 patients (Table 1) were collected for TEM imaging (Transmission Electron Microscopy), 60-90 days following the neovaginoplasty surgery.

**Table 1:** Results of Group 1 - characteristics of the group of patients with vaginal agenesis. Assessment of the level of intensity of the ERs on the neovaginas and electron transmission microscopy study.

Case	AGE (years)	MARITAL STATUS	COMPLAINTS	HEIGHT (cm)	WEIGHT	VAG. DEPTH BEFORE SURGERY	CHROMAT. GENE EXPRESS.	UROGRAPHY	FIRST SURGERY	COMPLICATIONS	VAGINAL DEPTH POST-SURGERY	ESTROGEN REC		
												DL	IL	SL
01	36	Single	Amenorrheic	154	48	Absent	+	Normal	McIndoe	-	8 cm	+++	+++	+
02	19	Single	Amenorrheic	155	54	Absent	+	Normal	McIndoe	-	5 cm	+++	++	+
03	21	Single	Amenorrheic	147	47	W/ 2 cm	+	Pelvic L kidney	McIndoe	Rectal lesion	6 cm	+++	+++	O
04	22	Single	without vagina	154	55	W/ 1 cm	+	Kidney and rounded/ bottom	McIndoe	Rectovaginal fist	6 cm	+++	+++	+++
05	28	Single	Without vagina	158	59	W/1 cm	+	Normal	McIndoe	-	7 cm	+++	++	O
06	17	Single	Painful intercourse			W/ 1 cm	+	Normal	McIndoe	-	8 cm	++	++	++
07	24	Married	Amenorrheic	163	58	Short	+	Normal	McIndoe	-	8 cm	+++	++	+
08	19	Single	Amenorrheic			W/ 2 cm	+	Normal	McIndoe	-	7 cm	+++	O	O
09	34	Single	Amenorrheic	152	55	W/ 1 cm	+	Normal	McIndoe	Rectovaginal fist	7 cm	+++	+++	+
10	19	Single	Amenorrheic	153	69	W/ 1 cm	+	Normal	McIndoe	-	8 cm	+++	+++	O
11	16	Single	Amenorrheic	165	47	W/ 2 cm	+	Normal	McIndoe	-	8 cm	++	O	+
12	22	Married	Amenorrheic	150	44	W/1 cm	+	Pelvic kidney	McIndoe	-	7 cm	+++	++	+
13	20	Married	Amenorrheic	160	44	W/ 2 cm	+	Pelvic Kidney	McIndoe	-	7 cm	+++	+++	+
14	20	Single	Amenorrheic	162	48	W/ 1 cm	+	Pelvic L kidney	McIndoe	-	7 cm	+	++	O
15	24	Single	Amenorrheic	158	44	W/ 1 cm	+	Normal	McIndoe	-	6 cm	+++	+++	O
16	18	Single	Amenorrheic	153	54	W/ 1 cm	+	Kidney and r cross o.	McIndoe	-	8 cm	+++	+++	O
17	28	Married	Without vagina		66	Absent	+		McIndoe	-	7 cm	+ + +	+ +	+
18	18	Single	Amenorrheic	163	54	W/ 1 cm	+	Dextrop l kidney	McIndoe	-	8 cm	+++	++	O
19	18	Single	Dyspareunia	146	58	W/ 1 cm	+	Ectopic l kidney	McIndoe	-	7 cm	++	++	+
20	23	Married	Amenorrheic	160	55	Absent	+	Pelvic l kidney	McIndoe	-	7 cm	+++	++	+
21	19	Single	Amenorrheic		48	W/ 2 cm	-	-	McIndoe+GON	-	6 cm	-	-	-
22	19	Single	Amenorrheic	165	57	Absent	-/46XY	-	McIndoe+GON.	-	7 cm	-	-	-
23	27	Single	Amenorrheic	160	67	W/ 1 cm	46XY	-	McIndoe+CLIT	-	4 cm	-	-	-
24	22	Married	Dyspareunia	158	45	W/ 2 cm	-	-	McIndoe +CLIT +GON	-	8 cm	-	-	-
25	18	Married	Dyspareunia	164	55	W/ 1 cm	-	Kidney and r cross o.	McIndoe+GON	-	7 cm	-	-	-
26	23	Single	Amenorrheic	161	49	W/ 1 cm	-	Normal	McIndoe+GON	-	8 cm	-	-	-
27	18	Single	Amenorrheic	160	46	W/ 1 cm	+	Normal	McIndoe	-	8 cm	-	-	-
28	18	Single	Amenorrheic	163	52	W/ 2 cm	+	Normal	McIndoe	-	7 cm	-	-	-
29	20	Single	Amenorrheic	166	58	W/ 2 cm	+	Normal	McIndoe	-	7 cm	-	-	-
30	21	Single	Amenorrheic	162	50	W/ 1 cm	+	Normal	McIndoe	-	8 cm	-	-	-
31	19	Married	Dyspareunia	160	68	W/ 1 cm	+	Normal	McIndoe	-	8 cm	-	-	-
32	24	Single	Amenorrheic	164	59	W/ 1 cm	+	Normal	McIndoe	-	6 cm	-	-	-
33	26	Single	Amenorrheic	167	57	VG W/ 3 cm	+	Normal	McIndoe	-	6 cm	-	-	-

### Manufacturing and preparation of the moulds and membranes

Molds manufactured during the preoperative period were made of artificial rubber sponge (synthetic latex), 14-16 cm in width and 3-4 cm in diameter, covered with a condom. The amniotic membranes were collected from patients during labor. Observing all asepsis requirements, chorion and amnion were separated after placental discharge, developing a rectangular 20x15 cm amnion fragment. This tissue was cleansed and covered with a moist dressing in a sterilized tray, containing 80 mg of gentamicin diluted in 100 ml of saline solution. Thus, the amnion collected was stored in a fridge under 4 degrees Celsius over a period of 48-72 hours before utilization. After the amnion membrane was collected, donators underwent serology studies to diagnose diseases like syphilis, toxoplasmosis, hepatitis, and HIV.

### Surgical technique

A 3 cm transversal incision was made in the vaginal introitus. A vesicorectal space was created by blunt dissection, creating a large cavity up to the Douglas pouch. The artificial rubber sponge mold, covered with a condom and amniotic membrane with its mesenchymal side externally arranged, was introduced in the vesicorectal space and the labia major was sutured using several cotton stitches in order to keep the mold with the amniotic membrane graft in place. After 7-8 days the mold with the condom were removed. A small-fragment biopsy (obtained from the lateral vaginal walls of this epithelium) was performed 60-90 days after the surgery, after metaplasia of the amniotic membrane into the vaginal epithelium had occurred. After a local injection of lidocaine anesthetic, a 0.5 cm^2^ fragment of this epithelium was dissected and immediately placed in a recipient filled with ethanol absolute. A second biopsy sample was collected, held to the same standard of care, fixed by immersion into 3% glutaraldehyde and referred for TEM (Transmission Electron Microscopy) imaging study.

### Estrogen receptors

To ensure proper interpretation of the findings obtained from the light microscope, the following systematization was settled, based on the color of the cytoplasmic granulations of the cells, according to the standards of the Biogenex Laboratories ^(Sternberger, 1979)^. Two site members evaluated each slide and cell staining was disposed in different grades:

-ABSENT: 0 (zero) / absent
-PALE-YELLOW COLOUR: + / discrete intensity
-YELLOW COLOR: ++ / moderate intensity
-YELLOW-BROWN COLOUR: +++ / strong intensity
-DARK BROWN COLOUR: + + + + / pronounced intensity

## RESULTS

In our study, we assessed a group of 33 patients, consisting of two subgroups. The first subgroup, A, included 27 patients with Rokitansky-Kuster-Hauser Syndrome and positive BARR sex chromatin pattern ^(Rokitansky, 1838)^. The second subgroup, B, included 6 patients with negative sex chromatin pattern and male pseudo hermaphroditism (Androgenic Insensitivity Syndrome/Morris Syndrome) ^(Morris & Maesh, 1963)^. The vaginal length was measured (vaginometry) before and after surgery (Table 1). Before surgery, the vagina was absent in 5 patients (15.15%), vaginal length was 1 cm in 19 patients (57.58%); and between 2-3 cm in 9 patients (27.27%). After surgery, all patients had vaginal length greater or equal to 5 cm and, in 26 patients (78.8%), the vaginal length was 7-8 cm, which brought them a satisfactory sex life.

### Transmission electronic microscopy of the vaginal neoepithelium following neovaginaplasty

**Figure 1:**
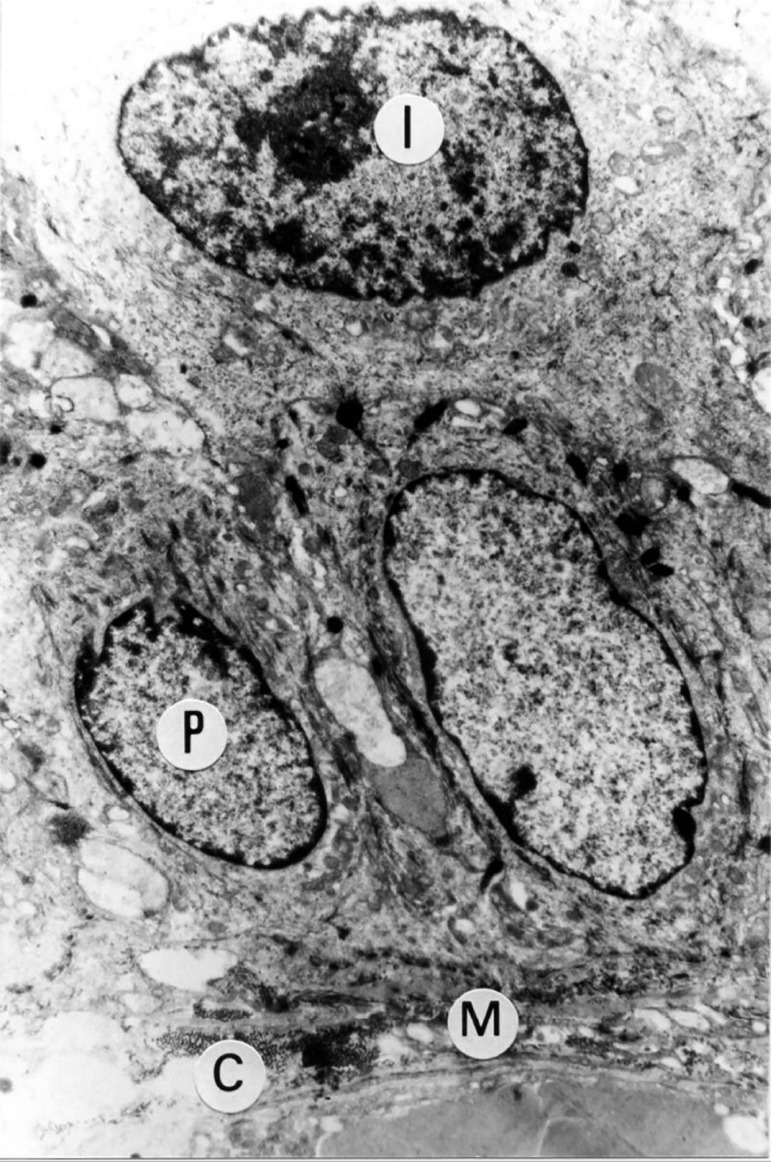
TEM of case No. 19, image showing ultrastructural appearance of neovaginal mucosal lining, similar to a normal mucosal lining, presenting chorion (C), basal membrane (M) and, in this epithelium, deep (P) and intermediate (I) layer cells are noted. Cytoplasmic and cell organelle characteristics are similar to those of a normal epithelium. Image size increased 7,300 fold.

**Figure 2:**
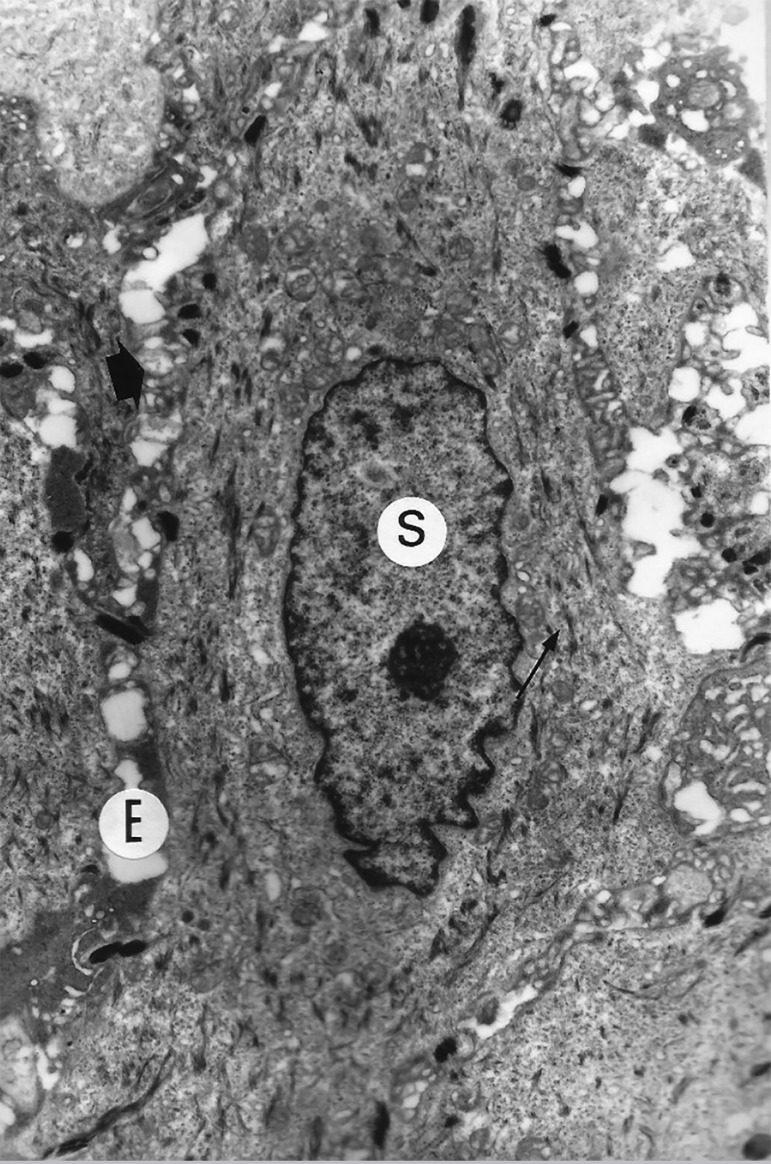
TEM of case No. 16, image showing the neovaginal mucosal lining, containing superficial cells (S), including desmosomes, cytokeratin filaments and intercellular spaces, identical to the normal vaginal epithelium. Image size increased 10,500 fold.

Ten patients who underwent neovaginoplasty were evaluated with Transmission Electron Microscopy (TEM) imaging (Table 1: cases No. 4, 14, 16, 17, 19, 27, 28, 30, 31, 33). The ultramicroscopy showed vaginal neoepithelium with normal mucosal lining, chorion, basal membrane and pavement-epithelium. Within this epithelium are deep, intermediate, and superficial layer cells, while the cytoplasmic and cell organelle characteristics are similar to those of a regular epithelium. Figures 1 and 2 are examples of the ultrastructural findings. TEM imaging showed no structures similar to the cuboidal epithelium found in the fragments of the amnion membrane graft in the neoepithelium lining. TEM morphological evaluation of the deep, intermediate and superficial layer cells showed a quite distinct nucleus x cytoplasm relationship. The nuclei in the deep layer are larger and have loose chromatin. In turn, the nuclear chromatin of the superficial layer cells is condensed. TEM analysis of the vaginal neoepithelium obtained from the amniotic membrane graft revealed all characteristics of a trophic vaginal epithelium.

### Estrogen receptors (ERs) determination

**Figure 3:**
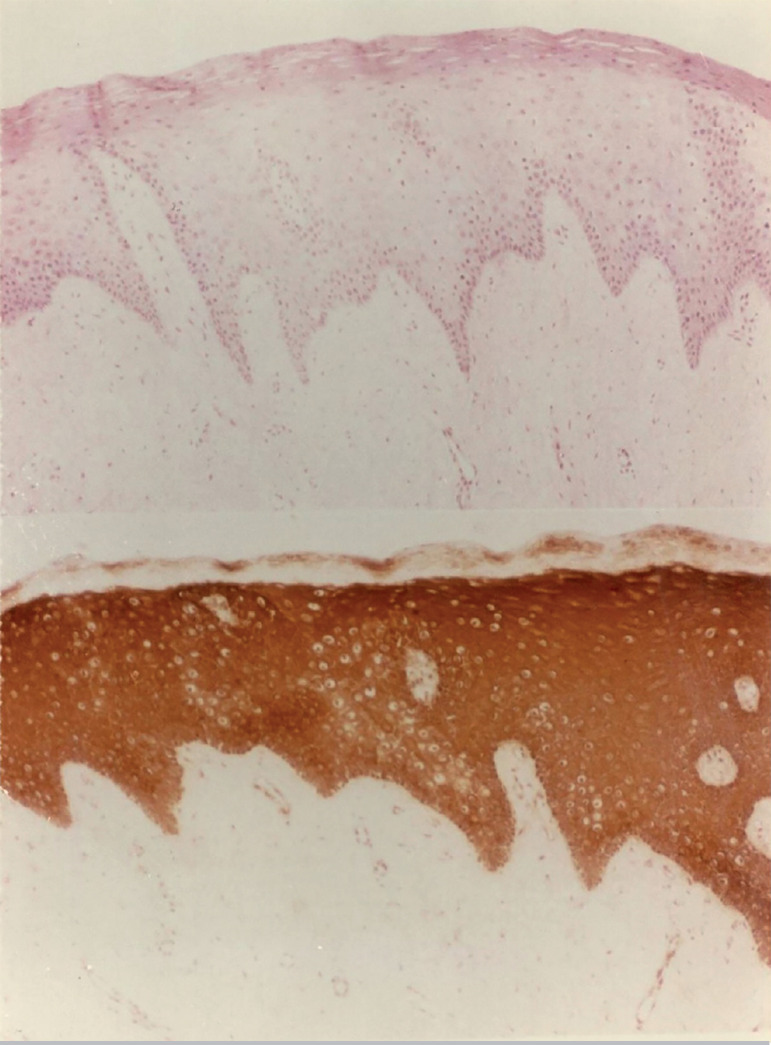
ERs determination in a histological slice of neovaginal biopsy - Table 1 - Case No. 6. Patient N.A.M., 17 years old, level of ERs intensity in the deep layer was ++ (2 crosses), ++ (2 crosses) in the intermediate layer and + (1 cross) in the superficial layer. Image size increased 100 fold.

This study included 26 patients; data are shown on Table 1. The ER determination in the epithelium of neovaginas was evidenced in variable levels of intensity, over different mucosal layers. They were subdivided in two subgroups. Subgroup A, included twenty patients with Rokitansky-Kuster-Hauser Syndrome, and Subgroup B, included 6 patients with male pseudo hermaphroditism, where ERs were not detected. The ERs presented different levels of intensity in the three layers of the mucosal lining. The ERs intensity level in the deep, intermediary, and superficial layers of the vaginal neoepithelium can be seen in Table 1 and Figure 3.

Figure 3 depicts the characteristics of these epithelial linings and the presence of ERs, showing image slices of the neovaginal fragment. Several layers in the vaginal mucosal lining are easily seen in these epithelial fragments, including cytosolic receptors of virtually identical intensity level.

## DISCUSSION

In recent years, patients treated for vaginal agenesis underwent different techniques in the search for a more convenient solution for this abnormality. All studies, aimed at obtaining a well-epithelialized, trophic vaginal canal with different tissues, suitable for a proper sexual activity. Improvements in the surgical techniques and satisfactory results for the patients with vaginal agenesis were achieved with the use of skin grafts, pioneered by ^McIndoe & Banister (1938)^. Later analysis of these patients revealed that just a partial process of skin transformation into mucosa occurred in the vagina. Permanent areas of epidermalization were observed inside the vaginal cavity, revealing thickened skin patches and other areas with hair growth. In addition, a delay in the healing process or even the occurrence of keloid was observed in the area where the skin graft was removed, usually the outer fascia of the thigh or gluteus. Later, we noticed that the amnion membrane graft was able to induce a neoepithelium growth in the recently opened vaginal cavity. ^Dhall (1984; 1987)^ and ^Ashworth *et al*. (1986)^ observed the amnion also presents favorable antigenic conditions with no membrane rejection.

Since 1985, ^Piazza & Teixeira (1992)^ have been pioneers in the use of amnion membrane fragments to correct vaginal agenesis and vaginal gynatresia in Brazil. Following this method, the modified McIndoe-Banister technique has been considered an easy and fast technical procedure. Human amnion is easily accessible, a good and biological dressing and may promote an excellent epithelization. ^Faulk *et al*. (1980)^ revealed microscopic evidence of new vessel formation and proposed that an angiogenic factor could be produced by the amnion. With the amnion employment, no immune rejection is developed for that membrane, it does not express histocompatibility antigens and the tissue aggregation is generally complete and presents no rejection. ^Akle *et al*. (1981)^ found no evidence of infections since the amnion has an antibacterial effect.

^Vatsa *et al*. (2017)^ performed an analysis in 50 patients with vaginal agenesis that were submitted to neovaginoplasty with amnion. All patients obtained successful results with vaginal length and sexually active life. Vaginal biopsy was done in four patients showing, after six months, a complete epithelization of the vaginal mucosa. ^Dornelas *et al*. (2012)^ performed neovaginoplasty in 11 patients with vaginal agenesis using a mold wrapped in an oxidized cellulose membrane. At 6 months, anatomical success was achieved in neovaginal length, and biopsy results showed complete epithelization of the neovagina. After 5 months, collagen content was comparable to that of a normal vagina. ^Kajikawa *et al*. (2012)^ performed immunohistochemical evaluation of the expression of estrogen receptor alpha on neovaginal tissue of patients with vaginal agenesis (Mayer-Rokitansky-Kuster-Hauser Syndrome) in serial samples obtained after surgery. A total of 22 samples of neovaginal tissue was analyzed in 3 groups and the ER alpha expression after 2, 3, 5 and 6 months of surgery. The conclusion revealed that the expression occurred when complete epithelization of vaginal tissue succeeded.

Transmission electron microscopy (TEM) studies including ultrastructural analysis were conducted aiming to register the transformation process underwent by the amnion. TEM-based amnion appearance showed that the epithelium is characterized by a single layer of cylindrical or cuboid cells resting over a chorion consisting of loose conjunctive tissue fibrils, which have a wavy appearance, parallel to the surface. We also noticed a complete transformation of the amnion epithelium, consisting of a single layer of cells, into a poliestratified, trophic epithelium, resembling a normal vaginal epithelium. Our purpose was to show the level of intensity of the cytoplasmatic determinant of the ERs in the different layers of this neoepithelium lining. Using those determinants, we were able to compare the level of intensity of the ERs in normal vaginas, according to ^Bleggi-Torres *et al*. (1997)^, and the group of patients with vaginal agenesis, where neovaginas were constructed using amnion membrane. Similar results were found in both groups. Human amnion contained multiple derived mesenchymal stem cells that are multipotent and able to differentiate into mesenchymal lineages, also being capable to develop into a polystratified vaginal mucosa ^(Moon *et al*., 2008)^. The mesenchymal stem cells are highly abundant on the amnion and, in the amniotic fluid; they can stimulate the development of a new vaginal mucosa ^(Fauza, 2004)^.

The wide variety of existing surgical techniques to correct vaginal agenesis leads to the conclusion that none of these techniques is ideal or offer no risks. This technical variant of the McIndoe-Banister surgery using amnion membrane grafts ensures achieving a deep, well-epithelized neovagina with good anatomical result.

## CONCLUSIONS

Surgical treatment of vaginal agenesis using amnion membrane grafts via the McIndoe-Banister technique enables the creation of a neovagina with a neoepithelium lining similar to that of a normal vagina.

The use of amnion membrane grafts to cover neovaginas shortens the surgical and post-procedural times.

The amnion tissue graft suffers a complete transformation evidenced by the TEM imaging.

The amnion membrane used to cover the neovagina undergoes a metaplasia process, resembling a normal vaginal mucosal lining, 60-90 days after the surgical procedure.

The levels of ERs intensity from the different layers of the vaginal epithelium, obtained from histological slices of women during menacme, resemble the histological slices resulting from biopsies of neovaginas covered with amnion membrane.
